# Influence of Submerged Arc Welding Current Intensity on the Mechanical Properties and Microstructure of Pressure Vessel P355N Steel

**DOI:** 10.3390/ma17143520

**Published:** 2024-07-16

**Authors:** Bogdan-Dorel Cioroagă, Ana Virginia Socalici, Vasile George Cioată, Emanoil Linul, Iosif Hulka, Iasmina-Mădălina Anghel

**Affiliations:** 1 Faculty of Engineering in Hunedoara, Politehnica University Timişoara, Revoluţiei 5, 331128 Hunedoara, Romania; bogdan.cioroaga@student.upt.ro (B.-D.C.); virginia.socalici@fih.upt.ro (A.V.S.); 2 Faculty of Mechanical Engineering, Politehnica University Timişoara, Blvd. M. Viteazu 1, 300006 Timişoara, Romania; emanoil.linul@upt.ro (E.L.); iasmina.anghel@student.upt.ro (I.-M.A.); 3 Research Institute for Renewable Energy, Politehnica University Timişoara, Str. Musicescu Gavril, nr. 138, 300501 Timișoara, Romania; iosif.hulka@upt.ro

**Keywords:** welding, seam, regime, structure, strength

## Abstract

This article presents a study on the influence of the intensity of the welding current on the properties of the mechanical strain strength of welded joints made by using submerged arc welding technology. The influence of the welding current on the microstructure of the welded joints was also observed in different regions of the cross-section of the welding seam. Also subject to observation was the mode of influence of the welding current on the geometry and dimensions of the welding seams. The welded samples were obtained using five different welding regimes whose main variable was the intensity of the welding current, which had values between 300 A and 700 A. The criterion used as a reference for comparing the effects produced by the studied welding regimes was a standard welding regime that is used in the industry for welding railway tank wagons, with a welding current intensity of 480 A. The base material used in the experiments was a fine-grained carbon steel specially intended for the manufacture of pressure vessels identified as P355 N; the semi-finished product from which the samples were made consisted of 6 mm thick laminated sheets used in the manufacture of the covers of the vessels that make up the railway tank wagon. The aim of this study was to highlight the differences that may appear through varying the current welding parameter and identify welding regimes that can provide welded joints with superior mechanical properties compared to what is currently employed in the industry. This study focused on the most widespread technology of welding pressure vessels: the submerged electric arc welding method.

## 1. Introduction

The manufacturing technology for pressure vessels (PVs) involves a series of processing materials specially designed to withstand the conditions of high pressure, temperature, and corrosion over time. A technological step with a large influence on the quality of the final PV product is the realization of welded joints for the manufacture of vessels. In the case of railway tank wagons, they are made in successive stages on large subassemblies such as chassis, bogies, and tank vessels and their assembly is accomplished both by welding and by means of assembly elements such as fasteners. PV design and technological aspects can be found in specialized literature, such as Ghanbari G et al.’s book [1] and the Moss D R series of PV manuals [2].

The welding methods used in the process of welding the components of railway tank wagons include several types depending on the area where the welding is performed; the chassis can be joined by shielded metal arc welding (SMAW) or by the MIG/MAG welding method with wire electrodes which can be both manual and automated for the subassemblies that allow this. Regarding the construction of tank wagon vessels, it requires the continuous welding of joints with long lengths that, in most cases, exceed 10 m. In order to be able to make these long welded joints, a welding technology used in the steel PV manufacturing industry involves arc welding covered with a layer of flux granules using a wire electrode (SAW). This welding technology is preferred because it offers high productivity, and the welding machine is a semi-automatic one, providing a constant value of the welding parameters throughout the welding process. More details about SMAW, MIG/MAG, and SAW welding technologies can be found in books, manuals, and specialized courses such as [3,4,5,6].

The body of the PV is made up of a middle area made of a laminated sheet covering, which was subjected to a rolling process to be curved, and later being closed by longitudinal welding. The other major component of the body of the PV are the heads which are made up of caps that were obtained from pressed sheet metal; they are welded to the central part of the container, forming a closed cylindrical capsule of a certain volume that is imposed by the design. The welded joint for joining the body of the container with the cup-type ends is made on a circular outline; the SAW process can only take place on the outside of the container and on its upper part to maintain the flow layer in the area of welding.

To determine an optimal welding regime that produces a quality of the joint superior to that currently achieved in the industry, it is necessary to understand the influence of the parameters that make up the welding regime. In the scientific literature, various studies have investigated the influence of certain parameters of the welding processes, taking into account various welding technologies. Researchers such as Yan Ma et al. studied how to improve the heat affected zone (HAZ) toughness of line pipes with thick walls using double-side SAW [7]. In the experiments, they used a commercial base material (X70) with a thickness of 28 mm, a diameter of 1422.4 mm, and contained a filler material of electrode wire with a 4 mm diameter. Metallographic analyses, Vickers micro-hardness analyses, and Charpy resilience tests were carried out on the welded specimens. Following the experiments, they made a series of findings: the CGHAZ toughness was negatively affected by a large PAGS and high HI and by rapid tempering or thermal treatment before welding; the ICCGHAZ toughness remains the same. Other research such as [8] where Han, Y. and others present aspects of welding defects and metal transfer that occurred during SAW processes; X-ray imaging was used to inspect the cavity and metal transfer evolution depending on the thickness of flux-cored wire electrode (FCAW) (1.6 mm, 19 mm, and 2 mm).

An approach to studying all the parameters involved in the submerged arc welding process involves successively varying one parameter or groups of parameters to observe the various effects on the quality of the welded joint. Various studies have addressed the problem of optimizing welding regimes, with the various process parameters variables as the input and the mechanical properties or microstructural changes of the material in the area of the welded joint as the output. An example of research that optimized these aspects is Pham Son Minh et al. [9], which presents an optimization of an orbital TIG welding process for welding SUS304 stainless steel pipes. They varied the welding angle, welding speed, welding current, pule time, and height of the torch; the base material consisted of pipes with a diameter of 76 mm and thickness of 1.5 mm made from SUS304 stainless steel, which was butt-welded using different values of the mentioned parameters. After the experiments, they found that the best quality of welding was obtained by using a 45 deg electrode angle; in order to achieve the ideal ultimate tensile strength, it was necessary to use a 45 deg electrode angle, 2 mm torch height, welding current of 174 A, 72 mm/min welding speed, and a 0.3 s pulse time. In order to determine an optimum welding process using gas metal arc welding (GMAW), Alnecino Netto, Francois Njock Bayock and Paul Kah in their research [10] used different GMAW parameters for welding Ultra-High-Strength Steel (UHSS). By performing a finite element analysis (FEA), different situations were simulated for the welding of S960 material plates; after analyzing the results, it was discovered that the welding parameters along with the heat input affected the microstructure of the UHSS.

The effects of the welding process parameters on the different base materials can also be evaluated by measuring the geometry and dimensions of the welded seam. Examples of research on how the geometry and dimensions of weld bead are affected include the research of Samir Khrais et al. [11] where they showed that GMAW process parameters impact the bead geometry and cause material distortion in a base material (AISI 316L) joined by butt-welding of two 100 × 100 × 5 plates using a V groove.

The joints made using various welding technologies can be subjected to tensile strength tests, resulting in stress–strain curves from which the tensile strength and other indicators that form the spectrum of mechanical properties can be determined. Based on the tensile strength, it can be determined whether the welded joints meet to the medium- and long-term operating requirements; their operating limit thresholds can be determined and specific safety factors for the operating situations of PV type products can be considered during the design phase of the product. By analyzing different welding regimes as well as different filler materials or basic materials for the welding process, an optimal solution can be determined in terms of the welding process and PV design. By testing several combinations of these conditions to obtain tensile test specimens, it is possible to obtain an overview of how the tensile strength and yield strength are influenced, which can be used in future PV design. Such experiments were carried out by many researchers, among whom, we can mention Aman Singh and R.P. Singh [12], who presented a literature review on the effects that the parameters of the welding regime have on the mechanical properties of assemblies joined using SAW technology. They highlighted the fact that increasing the welding voltage and current intensity leads to a decrease in impact resistance and tensile strength, while increasing the hardness. Experimental results that reflect and highlight the influence of the process parameters used in the SAW joint are presented in the research by Degala Ventaka Kiran and Suck-Joo Na [13]. They carried out experiments comparing the mechanical properties of different parameters for joints made by SAW from a single wire and from multi-wire welding. They concluded that the leading and trailing wire currents affect the strength and hardness of the welded joint.

Another method used on a large scale to determine the quality of welded joints is using a hardness probe at different points of the cross-section of the welded seam; this practice is used both in industry and in welded joint research centers where new joining methods are developed. By analyzing the hardness of welded samples with different welding regimes or different filler materials, it is possible to optimize the industrial processes through which the welded joints are made by determining the most favorable process conditions to obtain the desired quality in terms of mechanical properties. Masood Aghakhani et al. presented an analysis that considered the welding current intensity, arc voltage, and welding speed as the main variables along with other less common variables that are part of the SAW process and determined their effects on the hardness of the melted zone (HMZ) [14].

Investigations such as [15] showed the effect of welding parameters on the hardness of welded joints processed using MIG and SAW. The experiments carried out by Sumit Das Lala et al. in this study showed that a lower micro-hardness was observed in different zones in the welding seams produced by combined welding (MIG + SAW) compared with those produced using only SAW. A higher hardness was obtained for the combined welding process (MIG + SAW).

The optimization of the welded joint hardness using SAW by varying the welding current intensity, voltage, welding speed, and wire feed rate can be found in the research of Rudra Pratap Singh et al. [16]. The samples used in the study were 25 pairs of welded joints between 10 × 50 × 150 mm mild steel plates using different variations of the mentioned parameters.

In most research that records the study results of the influence of welding regime parameters include the changes that appear in the microstructure of the joint area. The use of different combinations of process parameters as well as the use of different values of the same parameter can have a significant influence on the microstructure of the welded joint. An example of a study that investigated the microstructural transformations caused by the SAW process in making the joints for the manufacture of pressure vessels is the research of Lochan Sharma and Rahul Chhibber [17], which showed the microstructures at 100× magnification to highlight that the fine-grained microstructure present in weld bead specimens had a basicity index that varied from 1.60 to 2.50.

Other authors such as Liangyun Lan et al. presented an analysis of SAW joints made of low carbon bainitic steel where microstructure variations were observed [18]. The specimens under study were made from 400 × 200 × 20 mm SAW plates, from which, a metallographic specimen and other test specimens were taken.

One study by Hüseyin Küçüköner et al., using P355GH base material from which SAW specimens were created, tested the specimens using magnetic particle inspection, radiography inspection, Charpy impact test, bending test, tensile test, harness test, fracture analysis, and optical and SEM analysis of microstructure [19].

This research analyzed the effects on the welded joint produced by certain variations in the value of the welding current intensity; the study was carried out using a set of five welding regimes that differ from each other only by the value of the welding current intensity. The standard submerged electric arc welding regime used in the railway tank wagon manufacturing industry served as the comparison to observe improvements or decreases in the quality of the welded joints. The aspects monitored in the experiments regarding the quality of the welded joints obtained using the different welding regimes under study were the transverse strain of the weld joint, microstructural changes in the joint area, micro-hardness distribution in the transverse cross-section of the weld, and variations in the geometry and dimensions of the welded seams.

The materials used in the experiments as well as the welding equipment were provided by the railway tank wagon manufacturing company Reva SA from the city of Simeria, Romania. The standard welding regime, which served as the reference for the other experimental regimes, is part of the serial manufacturing process used to manufacture the pressure vessels of the railway tank wagon produced by this company [20].

## 2. Materials and Methods

### 2.1. Materials

To carry out the experiments, 6 welded joints were made using submerged electric arc welding technology, each with a length of 300 mm; from each joint sample, 3 strain test specimens were extracted and a metallographic sample was taken from each of the 6 welding regime samples. The basic material from which the welded joints were made consisted of a 6 mm-thick laminated steel sheet semi-finished product of a fine-grained pressure vessel made of P355N steel (according to the standard EN 10027-1:2005 as it was a steel developed according to the standard SR EN 10216-3:2003) [21,22].

The filler material used was in the form of OK Autrod 12.24 electrode wire (according to the names given by its manufacturer ESAB, Hanover, PA, USA), which was a molybdenum-alloyed steel with copper clothing; it is a dedicated filler material for the submerged arc welding of pressure vessel steel.

The chemical composition of the welding base material (P355 N) (in maximum values) was as follows: carbon, 0.2%; silicon, 0.5%; manganese, 1.7%; phosphorus, 0.025%; sulfur, 0.02%; aluminum, 0.02%; chromium, 0.3%; copper, 0.3%; molybdenum, 0.08%; niobium, 0.05%; nickel, 0.5%; titanium, 0.04%; and vanadium, 0.1% [22].

The chemical composition of the welding filler material (OK Autrod 12.24) (in maximum values) was as follows: carbon, 0.12%; silicon, 0.2%; manganese, 1.2%; phosphorus, 0.02%; sulfur, 0.02%; and molybdenum, 0.6% [23].

The flux type material was named according to the manufacturer ESAB (Pennsylvania, USA), OK Flux 10.72. This is a powder-type material with a grain size of 0.315–2 mm. The role of this flux-type material is to provide a protective environment, presenting good insulation of the welded pool against atmospheric gases that can contaminate and oxidize the welded joint; at the same time, it offers an increased degree of safety for the operator of the welding machine and those around it by stopping the propagation of the light radiation of the electric arc and not allowing the gases from the molten bath to escape to the atmosphere. Two more benefits that the flux has on the welded joint can be mentioned, namely that it allows a slower cooling of the welded joint and contributes to the alloying of the welding pool with certain alloying elements that can normally be diminished by the gases emitted by the melted bath [24,25].

**Figure 1:**
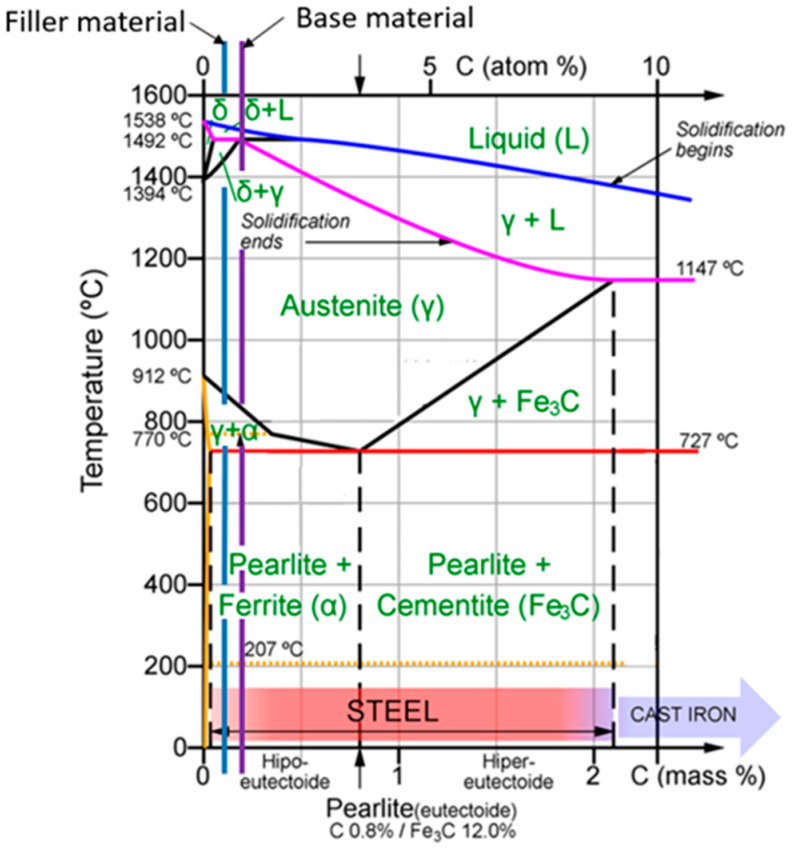
Base material and filler material on Fe_3_C steel equilibrium diagram [26].

Based on the chemical composition of the base material and the filler material, it can be seen that these are materials have a high degree of similarity to the alloying elements and most importantly, they have a similar carbon concentration. Figure 1 shows the placement of the two materials on the equilibrium diagram of the steels, where it can be seen that both materials are hypoeutectoid steels, which form ferritic and pearlitic structures under normal cooling conditions. In the case of accelerated cooling, considering the hypoeutectoid character of the welded joint, martensitic microstructures and bainitic microstructures may appear within the material in the joint area.

### 2.2. Manufacturing Process

#### 2.2.1. Manufacturing of Welding Joints

The welding machine used is a semi-automatic one that maintains constant values of the parameters of the welding regime throughout the process of joining the samples. The welding machine is composed of several parts, including a generic device found in most industries that make welded pipes from sheet metal semi-finished products that usually have large dimensions. The body of the welding machine is made of a mobile beam; on the end of the beam, the electrode holder torch is mounted, together with the drum with the electrode wire and the flux material management system. Near the mobile beam is the LAF 613-ESAB-type power source and the PEK-ESAB-type control unit from where the parameters of the electric arc and the travel speed are adjusted. The mobile beam can only make two types of movements, both in a straight line, one in the vertical plane and the other in the horizontal plane; the first movement brings the torch to the working level and the other is used for making the longitudinal welded joints.

The welded samples were made by butt-welding two 6 × 125 × 300 mm plates; the type of welding joint was type I with an interstice of 1 mm. The size of the plates that made up the samples were adjusted by guillotine cutting, and were later processed by grinding edges where the welding joints would be places to remove oxides and other impurities that could contaminate the welding bath. Keeping the welding joint in position during the welding process was achieved by manually executing two welding points at the ends of the joint; the plate obtained was clamped to the worktable using clamping devices. The welding was performed in a single pass in a horizontal position; the thickness of the flux layer deposited on the welding surface was approximately 25 mm [27,28].

Referring to the parameters of the standard welding regime that is used in the industry, we used the following constant parameters throughout the experiments:

- Welding current voltage: 33 V;
- Welding current intensity: 480 A;
- Welding speed: 60 cm/min;
- The tilt angle of the electrode: 90 deg.

**Figure 2:**
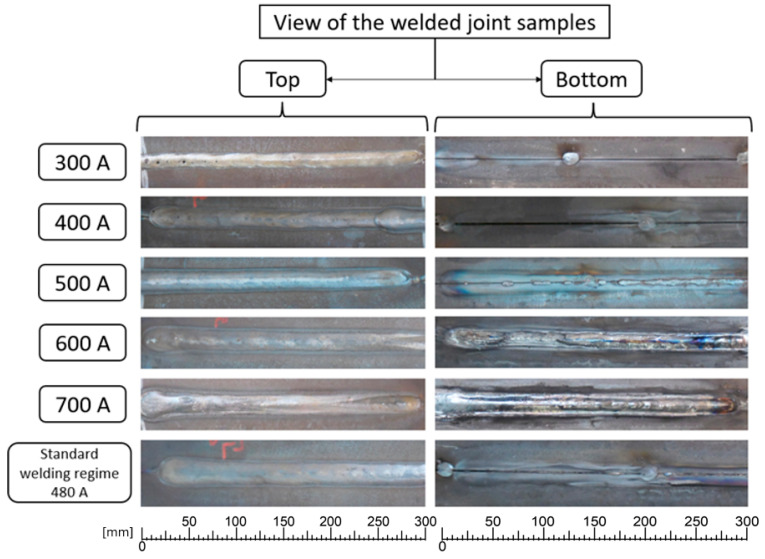
Samples welded using submerged arc welding with different values of the welding current intensity.

In addition to the standard welding regime made up of the previously mentioned parameter values, 5 more welding regimes were used, with different values for the intensity of the welding current: 300 A, 400 A, 500 A, 600 A, and 700 A. Figure 2 shows the welded samples obtained after the welding process.

It should be mentioned that for each set of samples, a ceramic support in the shape of a bar with a convex profile was used to support the molten material at the root of the joint so that it did not completely leak between the joints; this method is used in the current industrial process for railway tank wagons.

Figure 2 shows both the side of the sample on which the welding was performed (top) and the opposite side (bottom). The first observation was that the welding regimes with welding intensity values below 500 A had an incomplete penetration of the welding joint, and the filler material did not reach through the welding joint to the opposite side. Another observation that can be made from Figure 2 is that for values of the intensity of the welding current over 500 A, the bottom part of the samples showed a pronounced thermally influenced zone with a blue-green color [29].

#### 2.2.2. Manufacturing of Tensile Specimens

**Figure 3:**
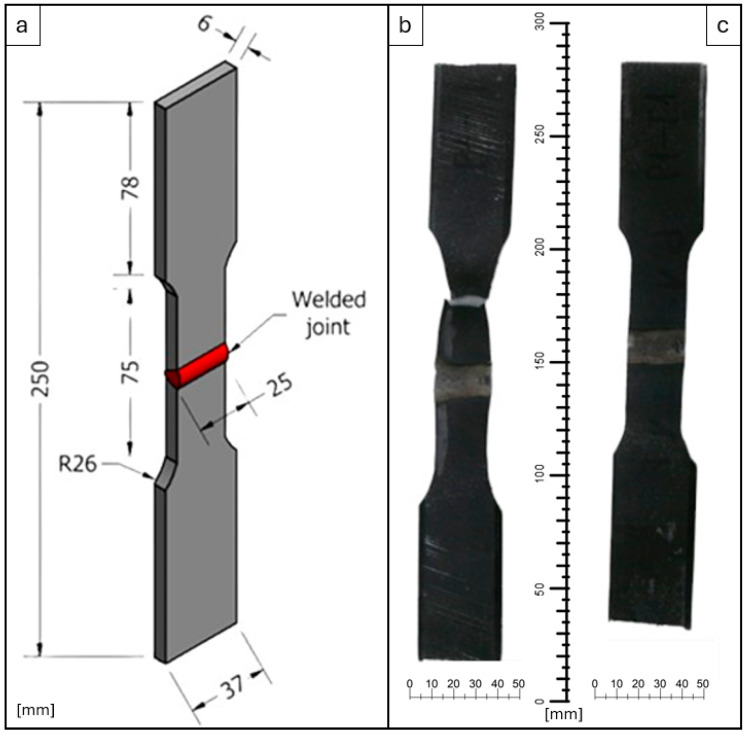
Geometry and dimensions of transverse strain test specimen. (**a**) 3D model of the specimen; (**b**) specimen after testing and (**c**) specimen before testing.

From the welded samples, 3 specimens from each sample were taken to test the welded joint under a transverse tensile load. The sampling of tensile test specimens was carried out in accordance with the standard “Destructive Tests on Welds in Metallic Materials—Transverse Tensile Test BS EN 895:1995” [30]. The processing of the tensile test specimens involved guillotine cutting to obtain the specified gauge and the final shape of the specimen was obtained by milling using oil cooling and a light chipping regimen involving high-speed small milling in order not to thermally affect the specimen’s material. The shape of the specimen and its dimensions are shown in Figure 3; all dimensions written in Figure 3a are in mm.

### 2.3. Experimental Setup

#### 2.3.1. Tensile Test

The ends of the specimen for tensile testing represent the areas where the specimen was fixed in the grips of the tensile testing machine; the middle area, which was narrower and where the welded joint was located, represents the area where the specimen will break. The machine used to carry out tensile tests was an A009 (TC100) universal testing machine, with a tensile capacity of 100 kN. The machine is composed of two clamps for holding the specimen; the lower clamp is fixed, and the upper one is mobile, which pulls the specimen with a gradually increasing stretching force until the specimen breaks.

The machine records every increase in the tensile force and every displacement movement of the mobile clamp; the recorded data were exported in the form of tabular values which were later processed resulting in stress–strain curves for each set of 3 specimens for each studied welding regime. From the stress–strain curves, a series of resistance characteristics of the welded joints can be determined, such as maximum force supported before breaking, maximum stress supported before breaking, strain at different stages of stress, yield tensile stress, and strain energy cumulated before braking [31].

The load was applied quasi-statically at 50 MPa/s. A transverse tensile force was applied to the welded joint in the middle of the test specimen inside the breaking zone. The test was stopped when a fracture occurred; two cases of fracturing are possible: through the base material or through the welded seam. The testing took place at room temperature.

#### 2.3.2. Hardness Test

The hardness measurements at different points of the cross-section of the welding seam was carried out using a 402 MVD hardness tester (Wolpert Group, Norwood, MA, USA) equipped with a Vickers intender. The sampling of the specimens for the Vickers hardness analysis took place by cutting the test specimens to a reasonable dimension; the cutting took place with continuous oil cooling and was made by sawing. After bringing the specimens to the desired dimensions, they were embedded in resin so that they could be polished using several sets of abrasive cloth until a smooth and flat surface was obtained.

The final shape of the test piece was round with two perfectly parallel faces, one of which was the polished face on which the measurements were made. Before the hardness testing, the samples were reacted with a reactive solution to highlight the HAZ. The Vickers hardness testing used controlled pressing of a pyramidal prism on different points of the samples. The hardness tester automatically displays the Vickers hardness values, which were collected and centralized for the construction of graphs of the changes in hardness in the cross-section of the welded seam.

#### 2.3.3. Microstructure

**Figure 4:**
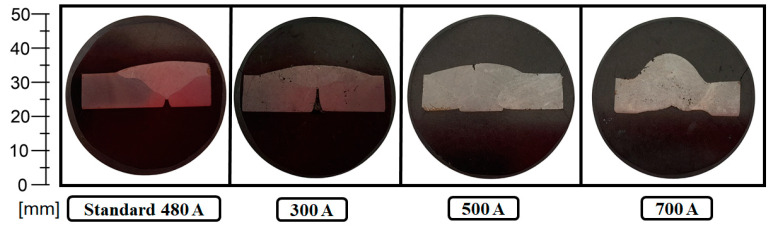
Samples for metallographic analysis of the microstructure of welded joints.

For the metallographic analysis, samples were taken from the specimens produced using the standard welding regime and the welding regimes with welding current intensity values of 300 A, 500 A, and 700 A. The metallographic samples prepared for the analysis of the microstructure of the welded joints are presented in Figure 4; the analysis was carried out by using a scanning electron microscope.

The preparation of the metallographic samples consisted of cutting operations using sawing and cooling oil in order to not thermally affect the microstructure of the sample. After obtaining samples with the correct size, they were embedded in resin tablets with parallel flat faces; one of the faces was polished by successively passing through several abrasive cloths (from 280 to 4000 grit) until a fine metallic mirror luster was obtained. In the last stage of processing, the polished surface of the metallographic sample was attacked with a Nital-type reagent to highlight the grain edges and the microstructure of the welded joint [32,33,34].

The microstructural examination of the specimens was carried out with the help of a scanning electron microscope (Quanta FEG 250, FEI, Hillsboro, OR, USA) using a secondary electron detector (SE) in high-vacuum mode at an acceleration voltage of 15 kV. The samples were prepared according to the Standard Guide for the Preparation of Metallographic Specimens, ASTM E3-11 [35].

## 3. Results and Discussion

### 3.1. Dimensional Analysis of Welds

**Figure 5:**
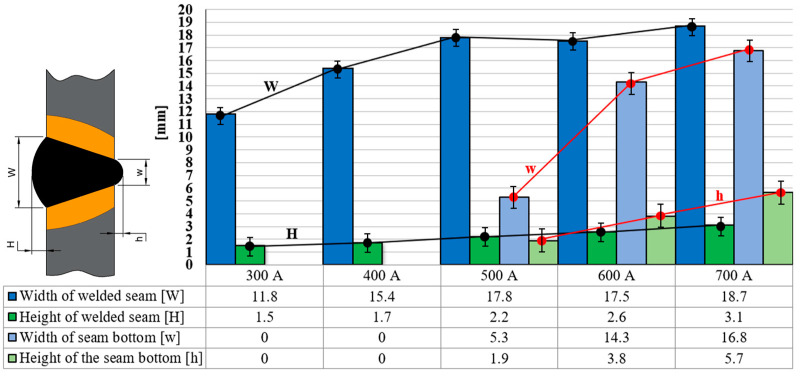
Welding seam dimensions H and W vs. the welding current intensity.

The results of the first study regarding the changes in the dimensional parameters that characterize the geometry of the welded joint are presented in the graph in Figure 5. Based on this graph, it is possible to notice a directly proportional correlation between the W, H, w, and h parameters and the value of the welding current intensity.

**Figure 6:**
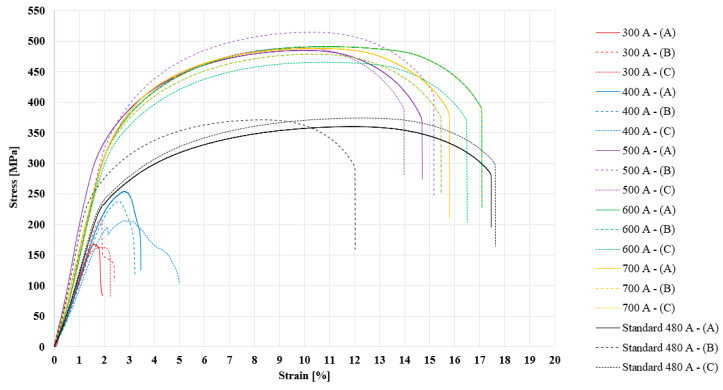
Stress–strain curves resulting from tensile testing of the welded specimens.

Comparing the numbers presented in the graph in Figure 6, the following average increases for the four dimensional parameters were found:

- Parameter W had an average relative increase of 13% for each 100 A, which translates to an absolute value of 1.73 mm for each 100 A.
- Parameter H had an average relative increase of 20% for each 100 A, which translates to an absolute value of 0.4 mm for each 100 A.
- Parameter w had an average relative increase of 47% for each 100 A, which translates to an absolute value of 4.2 mm for each 100 A.
- Parameter h had an average relative increase of 38% for each 100 A, which translates to an absolute value of 1.43 mm for each 100 A.

### 3.2. Tensile Results

Another set of data obtained was the stress–strain curves resulting from the tensile testing of the specimens taken from the welded samples. Figure 6 shows all of the stress–strain curves resulting from the experiments [31].

A clear differentiation can be observed between the different welding regimes in terms of the impact of the welding current intensity on the transverse tensile strength of the welded joint. Analyzing the graph obtained, it can be noted that the representative curves for the standard welding regime were in the middle of the range, with the lower border near the curves with a maximum stress starting from 180 MPa and the upper border was represented by the curves with a maximum in the area of 500 MPa. The fact that there were stress–strain curves with maximum stress values higher than the results obtained in the case of the standard welding regime indicates that there is room for improving the tensile demands of the welded joints by varying the welding current.

Judging based on the stress–strain curves, it can also be noted that the curves with a wide distribution along the strain [%] axis showed the characteristics of a welded joint with high ductility, and the curves with large areas under the curve can be characterized as being specific to welded joints with a high tenacity and storing a lot of energy before breaking.

Another important aspect is the nature and location of the specimen’s break, which indicates the sensitive area of the welded joint. We found the following characteristics of the tensile test specimen breakages:

- In the specimen set welded with 300 A, all three specimens suffered a break through the joint; the nature of the break was the detachment of the filler material.
- In the specimen set welded with 400 A, three specimens suffered a break through the joint; the nature of the breaks was the detachment of the filler material for one specimen and brittle fracture for two specimens through the filler material.
- In the standard welding regime set of specimens that were executed with 480 A, the break occurred through the base material in all three specimens, which were ductile fractures.
- In the specimen set welded with 500 A, the break occurred through the base material in all three specimens, which were ductile fractures.
- In the specimen set welded with 600 A, the break occurred through the base material for two specimens, which were ductile breaks, and in one sample, the break occurred through the filler material, which was a brittle break.
- In the specimen set welded with 700 A, the break occurred through the filler material for all three specimens; these breaks were all of a brittle nature.

**Figure 7:**
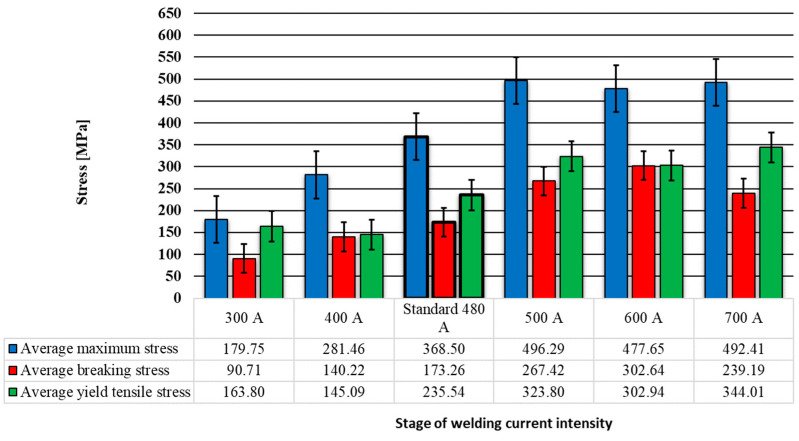
Average maximum stress results from tensile tests on welding specimens.

**Figure 8:**
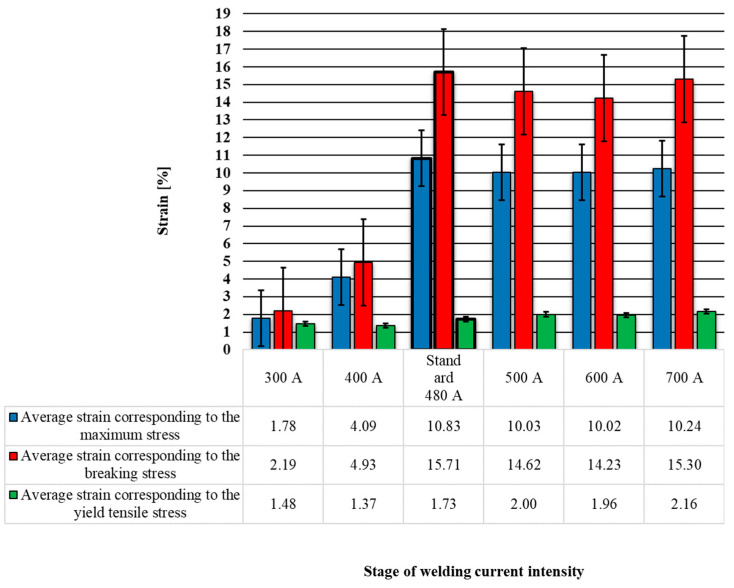
Average strain corresponding to the maximum stress results from tensile tests of welding specimens.

By processing the data obtained from the stress–strain curves presented in Figure 5, a set of strain strength parameters of the welded joint can be obtained, which are presented in Table 1. A graphic comparative representation of the changes in these strength parameters was extracted from this table and is presented in Figure 7 and Figure 8.

**Figure 9:**
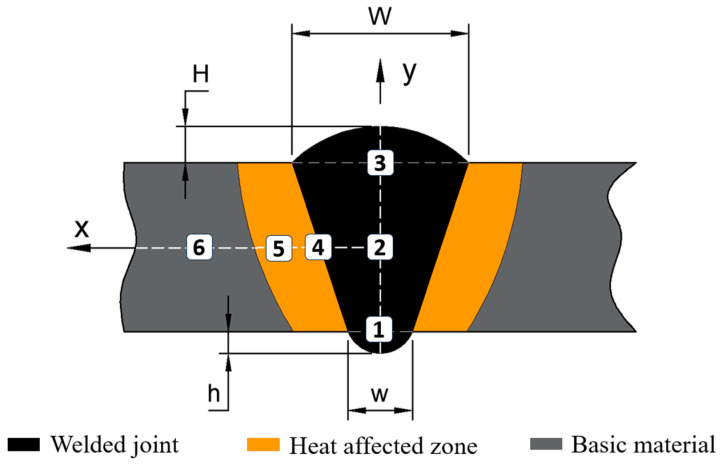
Analysis of the cross-section of the welded joint.

In Figure 7, where the main stress properties resulting from the tensile testing of the welded specimens are presented, all the stress properties can be noted as being directly proportional to the intensity of the welding current. The welding regime that produced the highest value of the average stress was the one with the welding current intensity of 500 A. Higher values than those presented by the standard welding regime regarding the stress that the welded joint can withstand were produced by all welding regimes with a welding current value of over 500 A. Taking the standard welding regime as a reference, we found the following observations of the three stress properties, which are shown in the graph in Figure 9:

- The tensile strength test parameter called average maximum stress, using 300 A, showed a decrease of 51% (−189 MPa); for 400 A, there was a decrease of 24% (−87 MPa); for 500 A, there was an increase of 35% (128 MPa); for 600 A, there was an increase of 30% (109 MPa); and for 700 A, there was an increase of 33% (124 MPa).
- The tensile strength parameter called average breaking stress, using 300 A, showed a decrease of 48% (−83 MPa); for 400 A, there was a decrease of 19% (−33 MPa); for 500 A, there was an increase of 54% (94 MPa); for 600 A, there was an increase of 75% (128 MPa); and for 700 A, there was an increase of 38% (66 MPa).
- The tensile strength parameter called average yield tensile stress, using 300 A, showed a decrease of 30% (−72 MPa); for 400 A, there was a decrease of 38% (−90 MPa); for 500 A, there was an increase of 37% (88 MPa); for 600 A, there was an increase of 29% (67 MPa); and for 700 A, there was an increase of 46% (108 MPa).

The three stress parameters are shown in the graph in Figure 7, where it can be seen that the trend for the changes in these stretching parameters was not similar to the one presented previously for stresses. Here, the standard welding regime produced the maximum values of the graph; the minimum values of the graph, similar to the graph in Figure 8, were produced by the welding regimes with welding current intensities of 300 A and 400 A. Also, the welding regimes with welding current values higher than 500 A showed high values of the stretching parameters (Figure 8), which were close to the values obtained in the case of the standard welding regime.

Taking the standard welding regime as a reference, we made the following observations of the three strain parameters shown in the graph in Figure 8:

- The tensile strength test parameter called average strain corresponding to the maximum stress, using 300 A, showed a decrease of −83%; for 400 A, there was a decrease of −62%; for 500 A, there was a decrease of −7%; for 600 A, there was a decrease of −7%; and for 700 A, there was an decrease of −5%.
- The tensile strength test parameter called average strain corresponding to the breaking stress, using 300 A, showed a decrease of −86%; for 400 A, there was a decrease of −69%; for 500 A, there was a decrease of −7%; for 600 A, there was a decrease of −9%; and for 700 A, there was a decrease of −3%.
- The tensile strength test parameter called average strain corresponding to the yield tensile stress, using 300 A, showed a decrease of −15%; for 400 A, there was a decrease of −21%; for 500 A, there was an increase of 16%; for 600 A, there was an increase of 13%; and for 700 A, there was an increase of 25%.

### 3.3. Hardness Results

Considering the dimensions shown in the sketch of the cross-section of welded joints presented in Figure 9, it is possible to assess the changes in the dimensional parameters that reflect the width of the top welded seam (W), the height of the top welded seam (H), width of the bottom welded seam (w), and the height of the bottom welded seam (h) depending on the intensity of the welding current. Also, based on the same scheme, the changes in the microstructure in different areas of the cross-section of the welding seam can be determined.

Zone 1 represents the root of the weld; for some samples, this area may be closer to the core of the weld bead or in line with the extreme line of the base material from the lower part of the weld bead, depending on the penetration rate of the filler material into the welding joint. The samples that were welded with welding current intensities higher than 500 A showed a zone 1 in line with the lower part of the base material, with a complete penetration into the root of the joint.

The area indicated with the number 2 on the vertical axis in Figure 9 represents the core of the welded joint and was placed on the axis of longitudinal symmetry of the base material which coincides with the x axis in the figure. Zone 3 mirrors zone 1 along the axis of symmetry; this represents the upper part of the welded seam that was in direct contact with the flux layer and where most of the filler material was deposited. Zone 4 represents the transition zone between the base material and the filler material, and is a mixing zone between the two materials; its position differs on the x axis from one sample to another depending on the width of the weld bead W and the degree of penetration of the filler into the base material. The last area that can be influenced by the value of the studied parameter, the intensity of the welding current, is zone 5, which represents the thermally affected area of the base material; the position of this area can differ on the x axis under the same conditions as for zone 4, but this area can cover a larger or smaller surface depending on the value of the welding current intensity.

Zone 6 does not have a variable character, being constant in terms of microstructure for all samples subjected to metallographic analysis.

**Figure 10:**
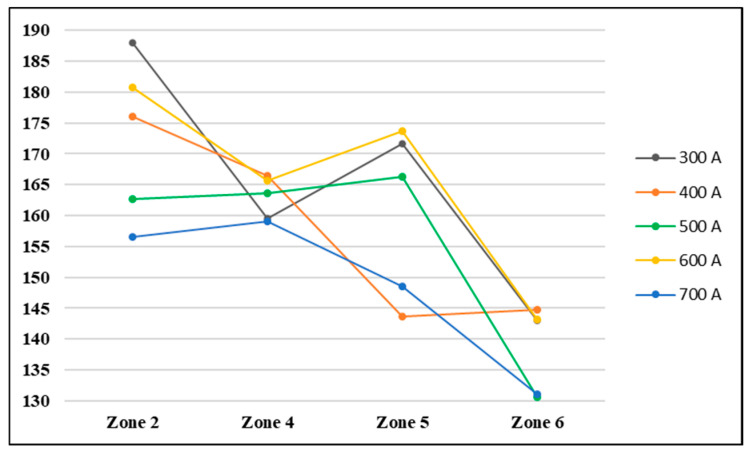
Hardness distribution in the cross-section of the weld on the x axis.

**Figure 11:**
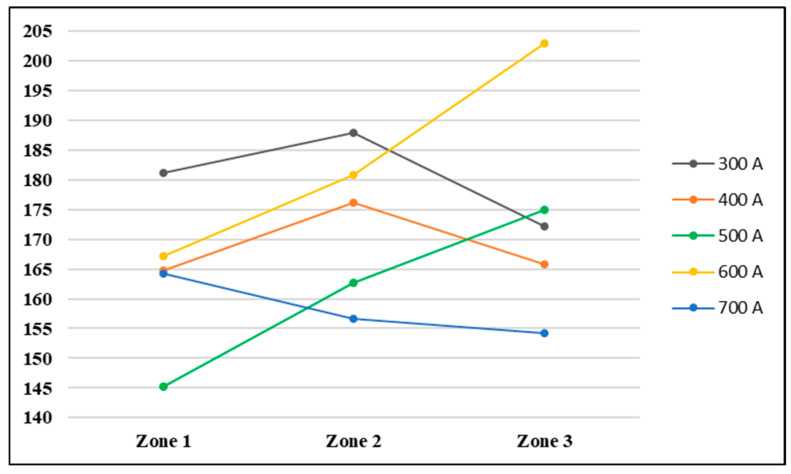
Hardness distribution in the cross-section of the weld on the y axis.

A complete picture of the changes in hardness in the six areas of interest mentioned previously is presented in the graphs in Figure 10 and Figure 11. The hardness analysis is presented by arranging the data collected from these areas from the cross-section of the welded seam on two distribution axes (x and y).

Observing the distribution of the hardness on the x axis from Figure 10, a general trend for all the welding regimes can be noted, in the sense that the highest value of hardness was recorded in zone 2 and the lowest in zone 6. The values recorded in zone 4 had the narrowest range of 158–167 HV. The widest intervals shown in the graph in Figure 10 were from zone 2 (156–188 HV) and zone 5 (144–174 HV).

The welding regimes that produced the highest hardness values according to the x axis distribution were those using welding currents of 300 A and 600 A and those that present the lowest values were those that use welding current values of 700 A and 500 A.

In the case of the hardness distribution on the y axis, according to the graph presented in Figure 11, two welding regimes showed an upward trend from zone 1 to zone 3; these are the welding regimes where 500 A and 600 A were used. The welding regime with a welding current intensity of 700 A showed a hardness distribution on the y axis with a decreasing trend from zone 1 to zone 3. The welding regimes produced a high general hardness on the y axis used 300 A and 600 A and those that produced the lowest values used 500 A and 700 A.

**Figure 12:**
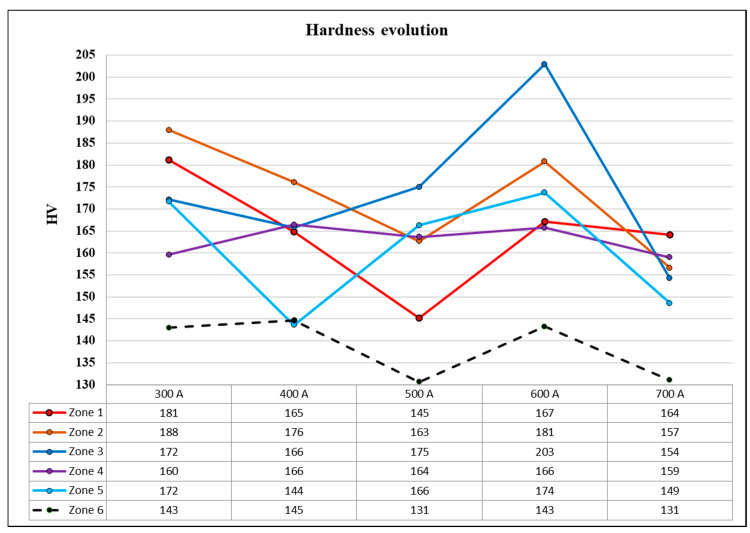
Hardness vs. welding current in different welding cross-section zones.

A more detailed analysis of the changes in hardness in the areas of interest depending on the value of the welding current intensity is presented with the help of the graph in Figure 12.

For zone 1, the hardness values showed a downward trend starting from the welding regime using 300 A up to 500 A, from where it begins an ascent from 145 HV to 167 HV for a welding current intensity of 600 A; the last point on the graph shows a slight decrease of 164 HV for the welding regime that uses a welding current value of 700 A.

It can be said that the hardness in zone 1 is inversely proportional to the welding current for welding current intensity values between00 A and 500 A, after which there is an increase followed by a maintenance level.

The changes in the hardness in zone 2 showed a downward trend with a jump from 162 HV to 181 HV for the welding regime that uses a current intensity value of 600 A. The hardness trend in zone 2 of the cross-section from the weld bead was identical to that of zone 1 in the range of 300–500 A.

Observing the graph of the changes in hardness in zone 3, it can be said that it starts with a small variation in the hardness between the regimes that use 300 A and 400 A; it can be considered to be a level zone and was followed by an ascending interval for the regimes of welding with welding current intensity values of 400 A to 600 A. On the ascending portion of the graph, a maximum of 203 HV was recorded and a sustained increase of 22% occurred throughout the ascending interval. The segment showing the upward trend was followed by a sudden decrease, registering the minimum point of the graph at 158 HV for the welding performed with an intensity of the welding current of 700 A.

Regarding the changes in the hardness in zone 3, it can be said that the interval 400 A–600 A had a similar trend to the other zones. The welding regimes found at the extremes of the graph did not follow the trends and their effects could not be predicted in terms of the hardness of zone 3.

The values of hardness recorded for the experiments in zone 4 showed a general oscillating trend with small oscillations. The minimum value recorded (158 HV) was from the welding regime that uses a welding current of 700 A, and the maximum value was produced by the welding regime with an intensity of the welding current of 400 A, with a hardness value of 166 HV.

Regarding the graph for zone 4, it can be said that it has very small variations, with an average value of 162 HV from the data from zone 4.

The changes in the hardness in zone 5 of the cross-section of the welded joints presented a central segment that includes the welding regimes with welding currents of 400 A, 500 A, and 600 A, which presented an upward trend. The minimum hardness value was recorded for the regime that uses 400 A (145 HV), and the maximum value was recorded for the regime with 600 A (174 HV).

The hardness values are shown in the graph in Figure 12 for zone 6, which consists of the base material that was not thermally affected by the SAW process. Data were recorded from each sample, from which it can be observed that there is an oscillatory trend. The minimum value of the graph (130 HV) was produced by the welding regime with 500 A and the maximum was 145 HV from the sample of the welding regime with 300 A.

### 3.4. Microstructure Analysis

**Figure 13:**
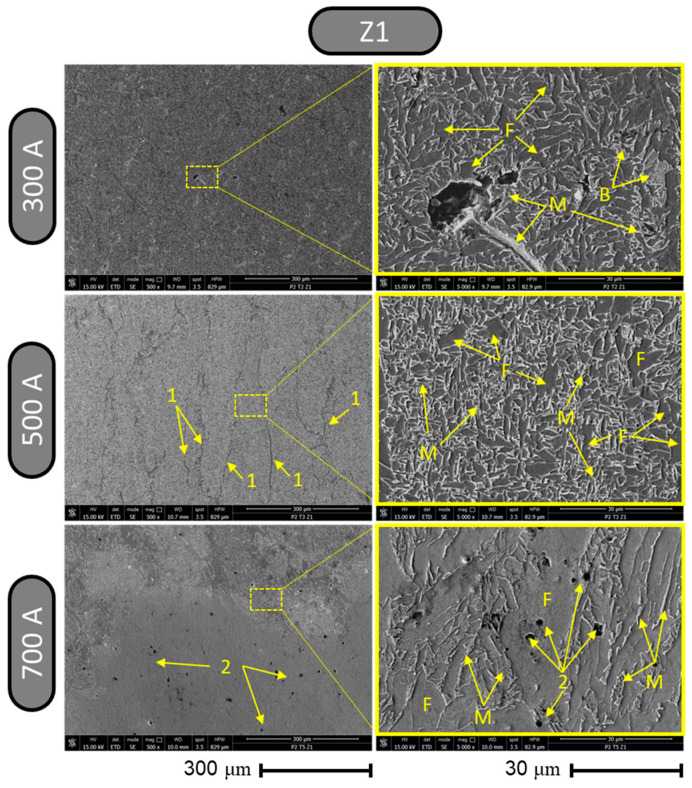
Results from metallographic structure inspection using SEM for zone 1.

The microstructure changes observed in the six zones of the cross-section of the weld indicated in Figure 6 were analyzed in comparative sets of images of three samples welded with regimes of 300 A, 500 A, and 700 A. The images collected using SEM from zone 1 representing the root of the welded joint are presented in Figure 13, where a uniform and homogeneous structure was observed at a magnification of ×500 using the welding regime with 300 A; using the welding regime with 500 A, a structure was obtained in which vertical ridges (labeled 1) were present that interrupted the homogeneity of the structure. In the case of the sample welded with 700 A, the presence of two regions was observed in zone 1; in the upper part, there was a region that presented a rougher appearance and in the lower part, a region that presented a smooth and dense appearance predominated. In both of these regions, there were dark points (labeled 2) that may represent the formation of different carbides or micro-cavities. Analyzing the surfaces at a higher magnification of ×5000, it can be seen that for the welding regime that uses 300 A, there were surfaces made up of ferritic phases (fine gray color labeled with F) that were bordered by martensitic (tagged with M) or bainitic (tagged with B) boundaries with a light-colored glossy appearance.

**Figure 14:**
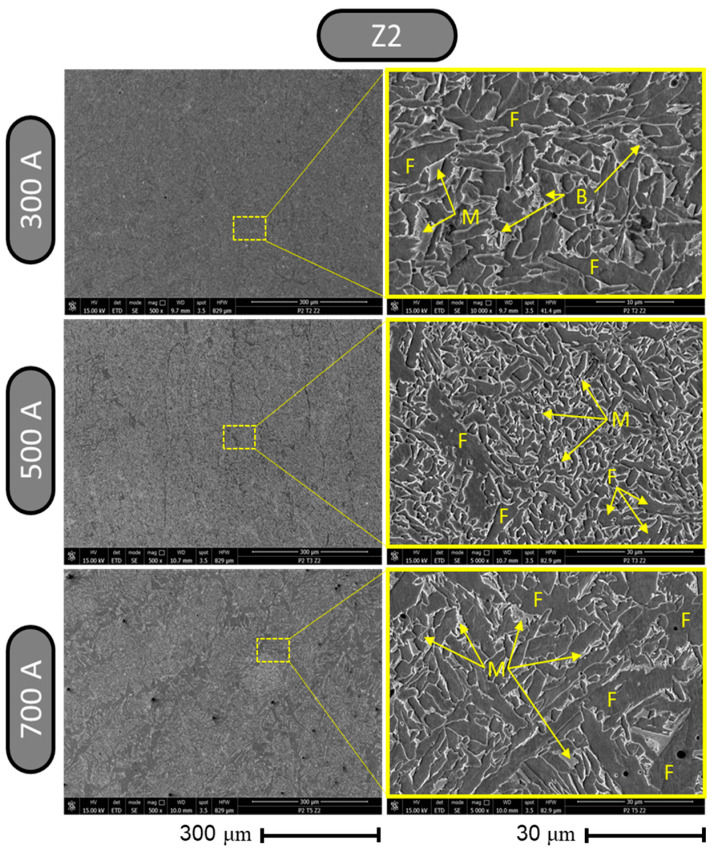
Results from metallographic structure inspection using SEM for zone 2.

Analyzing the changes in the microstructure in zone 2 depending on the intensity of the welding current (Figure 14), it can be noted that at a magnification of ×500, the structures did not show large differences; they were homogeneous, and a slight tendency of predominant ferritic phase stripes to appear was noticeable as the intensity of the welding current increased. At a magnification of ×500, the sample associated with the welding regime that uses 700 A showed a slight spread of some dark points that can be interpreted as carbides.

Observing the microstructure in zone 2 obtained after welding with 300 A using a magnification of ×5000, the formation of a structure made up of elongated ferritic surfaces (labeled F) in different directions was noticeable; these were bordered by a network of martensitic metallic bonds (labeled M), among which, bainitic formations (labeled B) also appeared. Also, at a magnification of ×5000, in the sample using a welding current of 500 A, there was a microstructure similar to the one presented in the case of the welding regime with 300 A; the only major difference was that for 500 A, the microstructure was finer with a predominance of martensitic phases (labeled M). In the case of the microstructure in zone 2 of the sample made by welding with 700 A, it was observed (at a magnification of ×5000) that there was a predominance of ferritic phases (labeled F), which presented as both small coverage regions and medium or large coverage regions.

**Figure 15:**
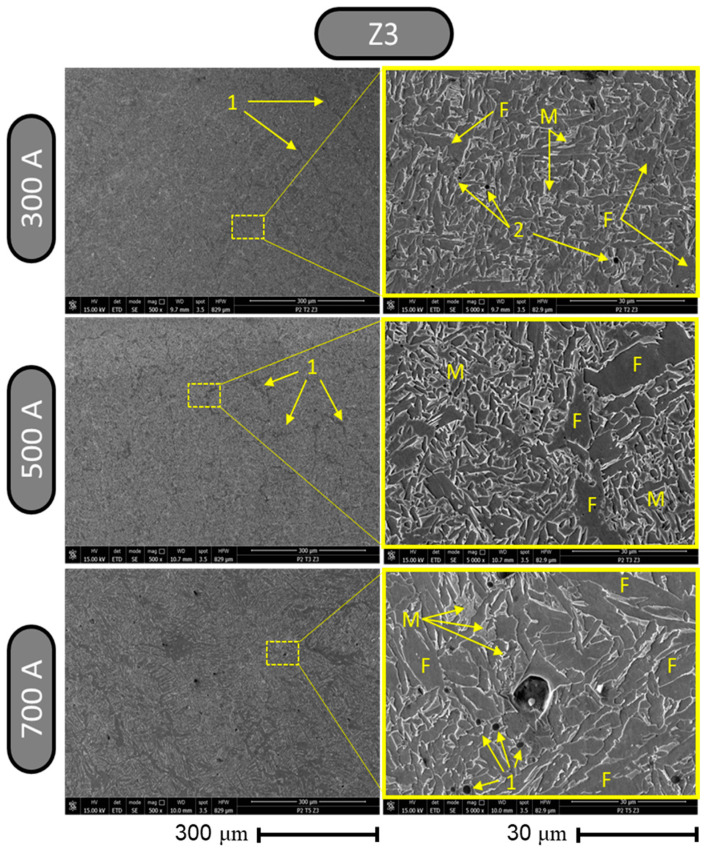
Results from metallographic structure inspection using SEM for zone 3.

The SEM images of zone 3 (Figure 15) showed, at a magnification of ×500, the development of a micro-topography consisting of fine stripes (labeled 1) for the welding regimes with 300 A and 500 A; for welding with 600 A, more pronounced stripes appeared along with the formation of dark points that may represent various types of microcarbs (labeled 2). Analyzing zone 3 in depth using a magnification of ×5000, it can be noted that the welding regime with 300 A produced a fine and homogeneous microstructure with small formations of microcarbs (labeled 2). The sample welded with 500 A showed, at a magnification of ×5000, a dense microstructure with pronounced edges and some prominent regions of ferritic phase formation (labeled F). The welding regime with 700 A showed (in zone 3 at a magnification of ×5000) a microstructure with thinner edges and a surface of ferritic formations (labeled F) that were much more predominant than in the other two previous cases; traces of carbide formation were also present (labeled 2).

**Figure 16:**
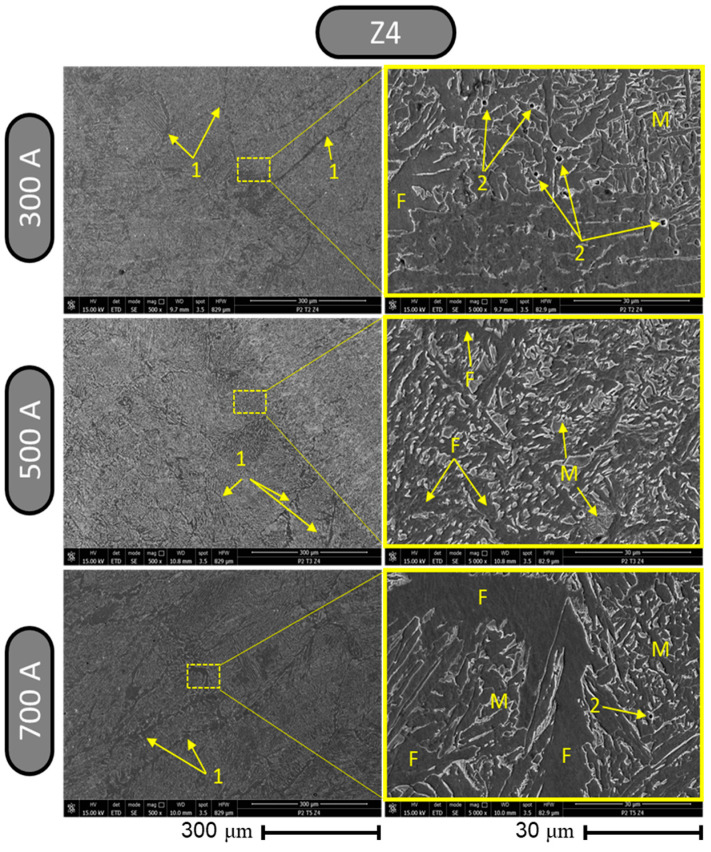
Results from metallographic structure inspection using SEM for zone 4.

Major differences regarding the changes in the microstructure appeared in zone 4, which can be observed in the set of comparative SEM images in Figure 16. At a magnification of ×500, a homogeneous and fine microstructure could be noted for all welding regimes, which, in the case of the sample made using 700 A, showed short parallel strips (labeled 1).

The analysis of zone 4 using a magnification of ×5000 highlighted different forms of the microstructure for each sample in terms of the form of the network. Welding with a current intensity of 300 A had the effect of forming a microstructure in zone 4 that presented a network with pronounced edges and some areas that did not form networks and were closed by edges that showed carbide formations (labeled 2) in some places. In the case of welding with a current intensity of 500 A, the formation of a microstructure with a globular appearance could be observed at a magnification of ×5000, which did not form regions closed by a clearly established border. The density of ferritic formations was lower than that of martensitic–bainitic transformation phases. In the case of the sample made with a welding current of 700 A at a magnification of ×5000, a microstructure was observed with large regions of ferritic phases (labeled F) and adjacent networks formed by both drop-type structures and closed structures, bordered by a pronounced contour of martensitic phases (labeled M).

**Figure 17:**
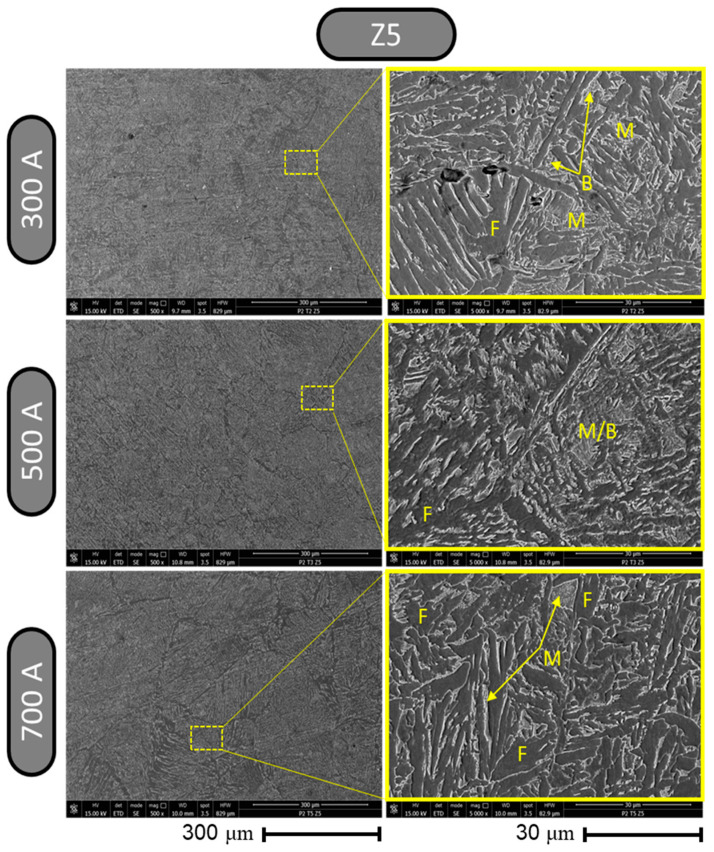
Results from metallographic structure inspection using SEM for zone 5.

The changes in the microstructure in zone 5 depending on the intensity of the welding current is shown in Figure 17, where a homogeneous structure with slight ripples can be observed at a magnification of ×500, which became more and more pronounced as the amperage value increased. At a magnification of ×5000, different shapes of the microstructure can be observed for the sample welded with 300 A; specifically, it was a mix of structures in the form of precipitation and structures in the form of a closed network. The sample welded with 500 A showed, at a magnification of ×5000, a structure in the form of a precipitate with a high density of martensitic (labeled M) and bainitic (labeled B) phases. The microstructure presented at a magnification of ×5000 in zone 5 in the sample made with a welding current of 700 A was in the form of a network with closed meshes.

**Figure 18:**
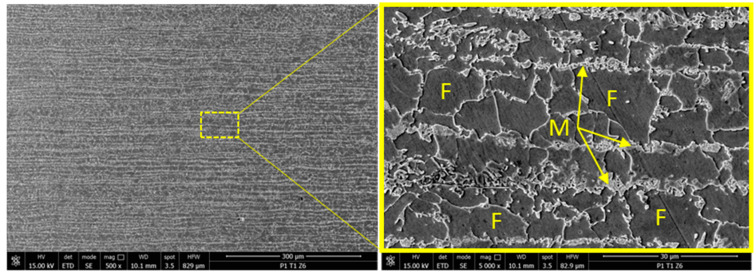
Results from metallographic structure inspection using SEM for zone 6.

The SEM analysis of the base material in zone 6 is presented in Figure 18 where, at a magnification of ×500, a lamellar microstructure can be observed showing parallel striations (labeled 1), all oriented in the same direction; these striations were associated with the mechanical process of laminating the semi-finished sheet. At a magnification of ×5000, a repetitive pattern of closed networks of similar sizes was visible, alternating with strips of curly structures that did not form closed meshes. The ferritic phases (labeled F) were predominant in this area, and are shown as a matte gray color.

## 4. Conclusions

- It was found after the visual inspection of the welded samples that the welds made with an intensity of the welding current of 300 A and 400 A showed an incomplete penetration of the filler material into the welding joints, while the welds made with higher amperage values showed a complete penetration.
- The analysis of the changes in the dimensional parameters of the welding found that the width W increased, on average, by 13% for every 100 A; the height H increased, on average, by 20% for every 100 A; the width w increased, on average, by 47% for every 100 A, starting from 500 A; and height H increased by 38% for every 100 A, starting from 500 A.
- From the tensile tests, it was observed that for the specimens welded using values of the welding current intensity of 300 A, 400 A, and 700 A, the rupture occurred through the weld seam, and for 500 A and 600 A, the rupture occurred through the base material. In the case of the samples made using 300 A and 400 A, breaking through the welding seam was favored due to the incomplete penetration of the filler material into the welding joints.
- The average stress indicators increased by between 30 and 35% for the welding regimes that use welding current intensity values above 500 A and for those with intensity values below 500 A, there were decreases of 24–54% compared to the standard welding regime used in the industry, which uses a welding current intensity of 480 A.
- The highest hardness was identified in the core of the welded seam (zone 2) with values between 158 and 188 HV. The lowest hardness values were recorded in the base material (zone 6) with values between 130 and 145 HV. The maximum hardness in zone 2 was obtained by using a welding current of 300 A, and the minimum hardness in zone 2 was recorded for the welding regime which uses a welding current of 700 A.
- Regarding the SEM analysis of the microstructure of the welding seam, it can be said that changes in the shape of the microstructural network were dependent on the intensity of the welding current; a microstructure with a denser network was noted for the welding regime using 500 A.
- Considering all the findings, it can be stated that an intensity of the welding current above 500 A produces welded joints with an increased resistance compared to those produced using the standard welding regime of 480 A. Regimes using currents below 500 A favor the appearance of welding defects such as a lack of penetration and result in the assembly breaking when stretched through the welded seam due to the detachment of the filler material from the base material.

## Figures and Tables

**Table 1:** Material strain strength parameters obtained from stress–strain curves.

Property	U.M.	Welding Current Intensity [A]					
		300 A	400 A	Standard 480 A	500 A	600 A	700 A
**Elongation**	mm	2.00	4.67	20.33	17.67	18.67	18.67
**Breaking force**	kN	42.09	65.11	85.91	86.38	85.61	85.51
**Maximum stress**	MPa	179.75	281.46	368.50	496.29	477.65	492.41
**Strain corresponding to the maximum stress**	%	1.78	4.09	10.83	10.03	10.02	10.24
**Breaking stress**	MPa	90.71	140.22	173.26	267.42	302.64	239.19
**Strain corresponding to the breaking stress**	%	2.19	4.93	15.71	14.62	14.23	15.30
**Yield tensile stress**	MPa	163.80	145.09	235.54	323.80	302.94	344.01
**Strain corresponding to the yield tensile stress**	%	1.48	1.37	1.73	2.00	1.96	2.16
**Modulus of elasticity**	MPa	85,311	141,240	145,953	223,873	234,526	125,920
**Fracture energy**	kJ	232	1025	4942	6118	5709	6435

## Data Availability

The original contributions presented in the study are included in the article, further inquiries can be directed to the corresponding author.
